# Identification and functional analysis of long non-coding RNA (lncRNA) and metabolites response to mowing in hulless barley (*Hordeum vulgare* L. var. nudum hook. f.)

**DOI:** 10.1186/s12870-024-05334-8

**Published:** 2024-07-12

**Authors:** Yixiong Bai, Jiaqi He, Youhua Yao, Likun An, Yongmei Cui, Xin Li, Xiaohua Yao, Shanshan Xiao, Kunlun Wu

**Affiliations:** Qinghai University, Qinghai Academy of Agricultural and Forestry Sciences, Qinghai Key Laboratory of Hulless Barley Genetics and Breeding, Laboratory for Research and Utilization of Qinghai Tibet Plateau Germplasm Resources, Xining, Qinghai Province 810016 China

**Keywords:** Hulless barley, lncRNA, Metabolome, Mowing, Plant hormones, Cytokinin

## Abstract

**Background:**

Hulless barley (*Hordeum vulgare* L. var. nudum Hook. f.) is a significant cereal crop and a substantial source of forage for livestock. Long non-coding RNAs (lncRNAs) and metabolites play crucial roles in the nutrient accumulation and regeneration of hulless barley plants following mowing. The study aimed to identify differentially expressed lncRNAs and metabolites in hulless barley plants by analyzing transcriptomic and metabolomic datasets at 2 h, 24 h, and 72 h following mowing.

**Results:**

The study revealed that 190, 90, and 438 lncRNA genes were differentially expressed at the 2 h, 24 h, and 72 h time points compared to the non-mowing control. We identified 14 lncRNA genes—11 downregulated and 3 upregulated—showing consistently significant differential expression across all time points after mowing. These differentially expressed lncRNAs target genes involved in critical processes such as cytokinin signaling, cell wall degradation, storage protein accumulation, and biomass increase. In addition, we identified ten differentially expressed metabolites targeting diverse metabolic pathways, including plant hormones, alkaloids, and flavonoids, before and after mowing at various time points. Endogenous hormone analysis revealed that cytokinin most likely played a crucial role in the regeneration of hulless barley after mowing.

**Conclusions:**

This study created a comprehensive dataset of lncRNAs, metabolites, and hormones in hulless barley after mowing, revealing valuable insights into the functional characteristics of lncRNAs, metabolites, and hormones in regulating plant regeneration. The results indicated that cytokinin plays a significant role in facilitating the regeneration process of hulless barley after mowing. This comprehensive dataset is an invaluable resource for better understanding the complex mechanisms that underlie plant regeneration, with significant implications for crop improvement.

**Supplementary Information:**

The online version contains supplementary material available at 10.1186/s12870-024-05334-8.

## Introduction

Hulless barley, also known as naked barley or qingke, is one of the earliest domesticated crops [1]. It is an important cereal crop in the Qinghai-Tibet Plateau region of China, where it has been cultivated for over 3500 years and serves as a staple food for the Tibetan ethnic group [2]. Hulless barley grains are highly nutritious and contain active compounds such as β-glucans, polyphenols, and tocopherols, which have therapeutic effects [3, 4]. This annual crop, used for both human consumption and animal feed, is noted for its adaptability to various environments, including cold and drought. Hulless barley plants develop rapidly, have a high tillering capacity, are highly regenerative, and produce much fodder. Green seedlings and straw from hulless barley are particularly valuable as supplementary feed in high-altitude areas [5]. Compared to other forages, hulless barley green seedlings are very palatable, high in sugars, and low in indigestible components like lignin and cellulose, which benefit livestock metabolism. Hulless barley straw, a vital renewable resource, is a primary raw material for straw feed, mitigating feed shortages to a certain extent [6].

Mowing is a highly effective approach to diversify crop utilization. When applied to dual-purpose crops, mowing provides valuable forage, improves crop efficiency, and reduces crop apical dominance by stimulating the production of new tissues and increasing the net photosynthetic rate [7]. Previous research has shown that mowing increases fresh weight production and the number of new leaves in plants [8, 9]. Furthermore, it improves plant productivity while maintaining plant community diversity [10, 11]. Decreasing mowing height reduces plant yield but increases nitrogen and phosphorus concentrations, indicating enhanced nutritional content [12]. Different mowing frequencies have a significant effect on plant growth and nutrient accumulation. For instance, a mowing frequency of four weeks resulted in the highest quality and regeneration capabilities of bermudagrass pasture [13]. Following mowing, significant changes occur in the starch and sucrose metabolism pathways, with increased degradation rates. Considerable alterations have also been observed in the metabolism of amino acid biosynthesis, phenylpropanoid biosynthesis, and the TCA pathway in nitrogen metabolism. Following mowing, stem growth accelerates while root growth is inhibited [14].

lncRNA plays diverse roles in plant growth and development [15, 16]. For instance, the lncRNA SABC1 regulates the expression of the transcription factor *NAC3*, affecting immunity and plant growth. LncRNAs can also influence plant growth by regulating the expression of genes involved in auxin synthesis and microRNA production. Wheat harvesting leads to increased expression of genes encoding enzymes such as β-amylase, sucrose synthase, sucrose-6-phosphate synthase, trehalose-6-phosphate synthase, and trehalose-6-phosphate phosphatase, indicating that carbon accumulation promotes plant regeneration. Differentially expressed lncRNAs may regulate these genes and hormone-related pathways [17]. Harvesting also leads to changes in nutrient accumulation. Plant regeneration is accompanied by significant metabolite changes, including a decrease in saturated fatty acids and a considerable increase in unsaturated fatty acids, which suggest changes in nutrient composition [18]. The early-stage harvesting of hulless barley, a crucial cereal crop, stimulates plant growth and boosts animal feed availability. However, the regulatory mechanisms of lncRNAs and metabolite alterations after harvesting are unknown. This study integrates transcriptome and metabolome datasets from hulless barley harvested at 2 h, 24 h, and 72 h after mowing to elucidate the molecular mechanisms underlying lncRNA regulation of plant growth and metabolite alterations. It provides molecular evidence of plant regeneration and changes in nutrient composition following hulless barley harvest.

## Materials and methods

### Plant materials and samples collection

Kunlun 18 (KL18) was grown in the experimental field (101° 74’E, 36° 56’N) of the Crop Institute of Qinghai Academy of Agriculture and Forestry Sciences in Xining, Qinghai Province. The test area is 2261 m above sea level and characterized by a continental plateau semi-arid climate marked by low rainfall, high evaporation, and significant diurnal temperature fluctuations. Stubble samples were collected after mowing at 0 h, 2 h, 24 h, and 72 h, with three replicates per time point.

### RNA extraction, transcriptome assembly, and qRT-PCR analysis

Tissue samples were instantly frozen in liquid nitrogen after collection from the field. Total RNA was extracted using TRIzol reagent (TransGen Biotech, Beijing, China) per the manufacturer’s instructions. Approximately 2 µg of total RNA was reverse transcribed using the Superscript III reverse transcriptase kit (Invitrogen, California, USA) following the manufacturer’s protocol to generate the first strand of cDNA. qRT-PCR experiments were performed on an ABI 7500 system (Thermo Fisher Scientific, California, USA) with FastStar Universal SYBR Green Master (ROX) kits (Roche, Basel, Switzerland). The relative expression was calculated, with the actin gene serving as an internal reference [19]. The primers used in the experiment are listed in Table 1. Network flow algorithms and optional de novo assembly were applied to assemble complex datasets into transcripts. Initially, pairs of overlapping reads were assembled into super-reads, which were then matched to the reference genome to construct a graph representing splice sites. A final assembly was performed by retaining paths with high read coverage to generate transcripts.

**Table 1:** Primers design for 12 mRNA target genes

Number	geneID	Genename	Forward primer sequence (5'-3')	Reverse primer sequence(5'-3')
1		18SrRNA-1	ACACTTCACCGGACCATTCAA	CTACGTCCCTGCCCTTTGTACA
2	rna-XM_045098651.1	LOC123404704	TGCGGCTGGACGGGATG	TGGCGTTGGAGATGGAC
3	rna-XM_045124790.1	LOC123448023	AAAGGGAACCGATGAT	ACACTTGTGGCTTACTGG
4	rna-XM_045094466.1	LOC123400068	GTTTCTGCGGCTTCT	TCTTTCTTATCGGCTTG
5	rna-XM_045101713.1	LOC123408654	GCGTTCCACTGCCACA	GCTCCCGCATCTTGTCT
6	rna-XM_045100912.1	LOC123407718	GTAAAAGCCACACCCA	GCCAGTATCTCCCCAT
7	rna-XM_045111976.1	LOC123427840	CTGGATACTTCGGCCGAGAC	TCGTGCTGTTGATCTGCCTT
8	rna-XM_045112522.1	LOC123428326	AGACCTTCCAACCCAACT	GTCCACAAACCCTCCATAC
9	rna-XM_045104931.1	LOC123411970	ACCTCGCCAACGGTATCTTG	ATATGTCGAGCTTCACC
10	rna-XM_045105407.1	LOC123412458	GCCAAACACCACATCACCAC	CGAACCCGATCCGATCTGAG
11	rna-XM_045104845.1	LOC123411884	ATAGTGCGACATCTGGT	GCTTTGGCGAAGTAGG
12	rna-XM_045114173.1	LOC123430303	CCTGACATCGTCCCTAC	TGCTTGGCTAAAGAAAT
13	rna-XM_045105116.1	LOC123412177	CTGGCGAGCGTGAGGA	CGGCGACGATGAAGTA

### Identification of long non-coding RNAs

We retrieved reported lncRNA sequences from lncRNA-related databases, including NONCODE, GreeNC, Ensembl, and NCBI, and identified known lncRNAs using these resources. The prediction of novel lncRNAs involves two main steps: basic filtering and coding potential screening. The steps include selecting transcripts with a length ≥ 200 bp and at least two exons, calculating the read coverage of each transcript using StringTie and selecting transcripts with a minimum coverage ≥ 3, comparing the transcripts with known non-lncRNA and non-mRNA types (such as rRNA, tRNA, snRNA, snoRNA, premiRNA, and pseudogenes) using cuff-compare and removing transcripts that exhibit similarity or identity to the aforementioned known transcripts. The transcripts were compared with known mRNAs and classified as candidate lincRNAs, intronic lncRNAs, antisense lncRNAs, or sense-overlapping lncRNAs, followed by advanced screening by assessing the coding potential of the initially filtered lncRNAs. We used CPC, CNCI, CPAT, and pfam protein domain analysis to intersect and screen lncRNAs.

### Extraction of metabolites

Following field sampling, tissue samples were immediately frozen in liquid nitrogen with three replicates per sample. The samples were ground with liquid nitrogen, and 100 mg were transferred to a centrifuge tube containing 500 µL of extraction solution (methanol–water, volume ratio 3:1, with internal standard). The mixture was vortexed for 30 s before adding two small steel beads and grinding at 45 Hz for four minutes. Ultrasonication was performed in an ice-water bath for 15 min. After overnight shaking at 4 °C, the mixture was centrifuged at 4 °C at 12,000 rpm for 15 min. The supernatant was filtered through a 0.22 µm filter membrane. The filtered samples were placed in 2 mL injection vials, and 30 µL of each was combined to create a quality control (QC) sample. The target compounds were chromatographically separated using the EXION LC System (SCIEX) ultra-high-performance liquid chromatography instrument with a Waters UPLC column. The mobile phase A consisted of a 0.1% formic acid aqueous solution, while mobile phase B comprised acetonitrile. The column oven and the autosampler temperatures were set at 40 °C and 4 °C, respectively. The injection volume was 2 µL. Mass spectrometry analysis was conducted using the SCIEX 6500 QTRAP + triple quadrupole mass spectrometer equipped with an IonDrive Turbo V ESI ion source. The ion source parameters were as follows: Ionspray Voltage: + 5500/-4500 V; Curtain Gas: 35 psi; Temperature: 400 °C; Ion Source Gas 1: 60 psi; Ion Source Gas 2: 60 psi; and DP: ± 100 V.

### Determination of endogenous hormones following mowing

The KL18 and Z1257 varieties were planted in the experimental field (101° 74’E, 36° 56’N) at the Crop Institute of Qinghai Academy of Agriculture and Forestry Sciences in Xining, Qinghai Province. After mowing, stubble samples were collected at 0 h, 2 h, 24 h, and 72 h, with three replicates per time point. For hormone extraction, an isopropyl alcohol/water/hydrochloric acid solution was used, with acid added to enhance the hormone solubility in the organic solvent and inactivate certain enzymes in the tissue. The samples were concentrated using dichloromethane extraction, then analyzed with HPLC–MS/MS and quantified using an external standard method. The extraction process began by weighing 0.1g freshly ground plant material into a test tube and adding 1 mL of extraction solution to each tube.

We used a 1:10 material-to-liquid ratio and placed the sample in a shaker at 4 °C in the dark for 30 min. The mixture was then treated with 2 mL of dichloromethane so that the ratio of extraction solution: dichloromethane = 1:1 ~ 1:2. The resulting solution was shaken at 4 °C in the dark for 30 min. The solution was centrifuged at 4 °C, 13,000 rpm for 5 min. After centrifugation, two phases formed with plant debris between the two layers. We carefully transferred the upper phase to a centrifuge tube containing 0.2 g of anhydrous magnesium sulfate. The mixture was vortexed for 1 min and then centrifuged. The resulting supernatant was transferred to a new 10 mL centrifuge tube equipped with a cover, keeping it away from light. The samples were dried in a centrifugal concentrator at a temperature below 30 °C. Once dried, the samples were reconstituted in 0.2 mL of a 0.1% formic acid–methanol solution and detected by HPLC–MS/MS.

## Result

### Identification of long non-coding RNAs in hulless barley

**Fig. 1:**
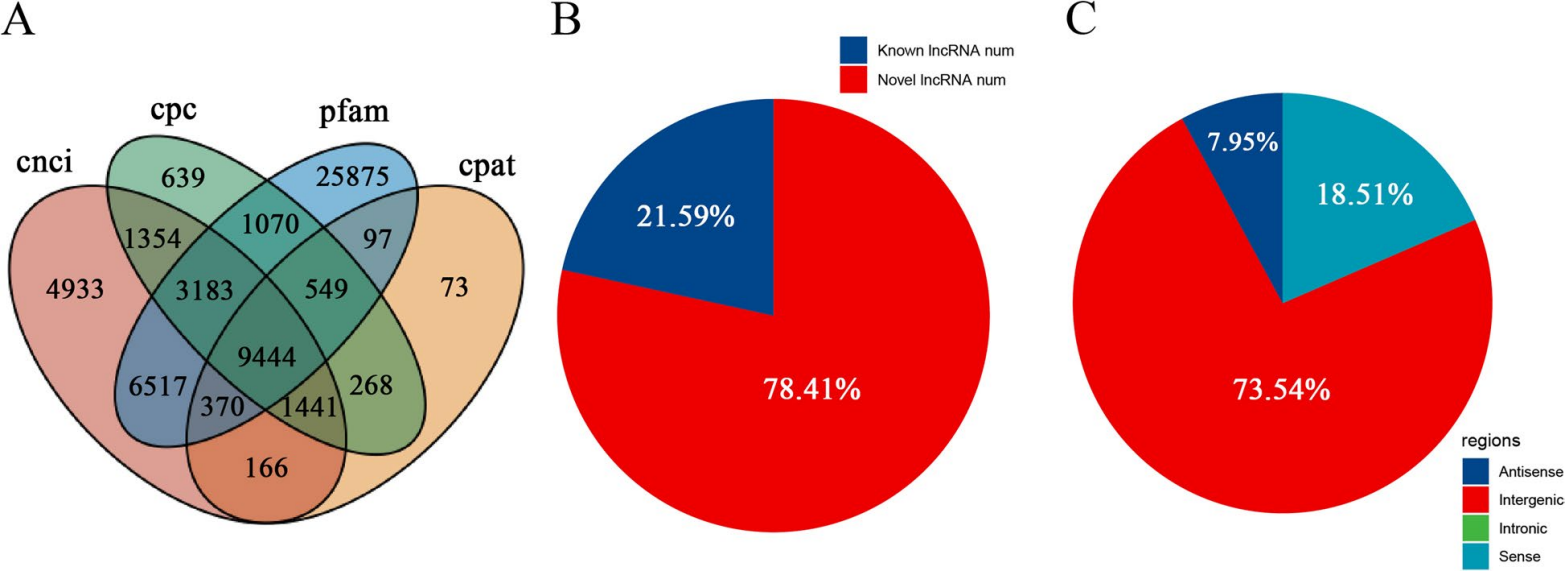
The identification and classification of lncRNAs in hulless barley.  **A** Various analytical methods used to identify lncRNAs. **B** Category of lncRNAs. **C** Classification of lncRNAs

We used transcriptome sequencing on stubble samples collected at 2 h, 24 h, and 72 h to thoroughly examine the landscape of non-coding RNAs (lncRNAs) in hulless barley following mowing. We assembled the transcriptome and identified, characterized, and analyzed the lncRNAs. The RNA-seq reads were aligned to the reference genome of hulless barley, and data was assembled. Rigorous criteria were used to identify lncRNAs (as detailed in the Materials and Methods section). As a result, 9444 novel lncRNA genes were identified (Fig. 1A, B), with a high proportion of these being new. Intergenic lncRNAs exhibited the highest count, while intronic lncRNAs had the lowest. Fig. 1C shows that both antisense and sense lncRNAs were in the intermediate range.

### Characteristics of novel lncRNAs

**Fig. 2:**
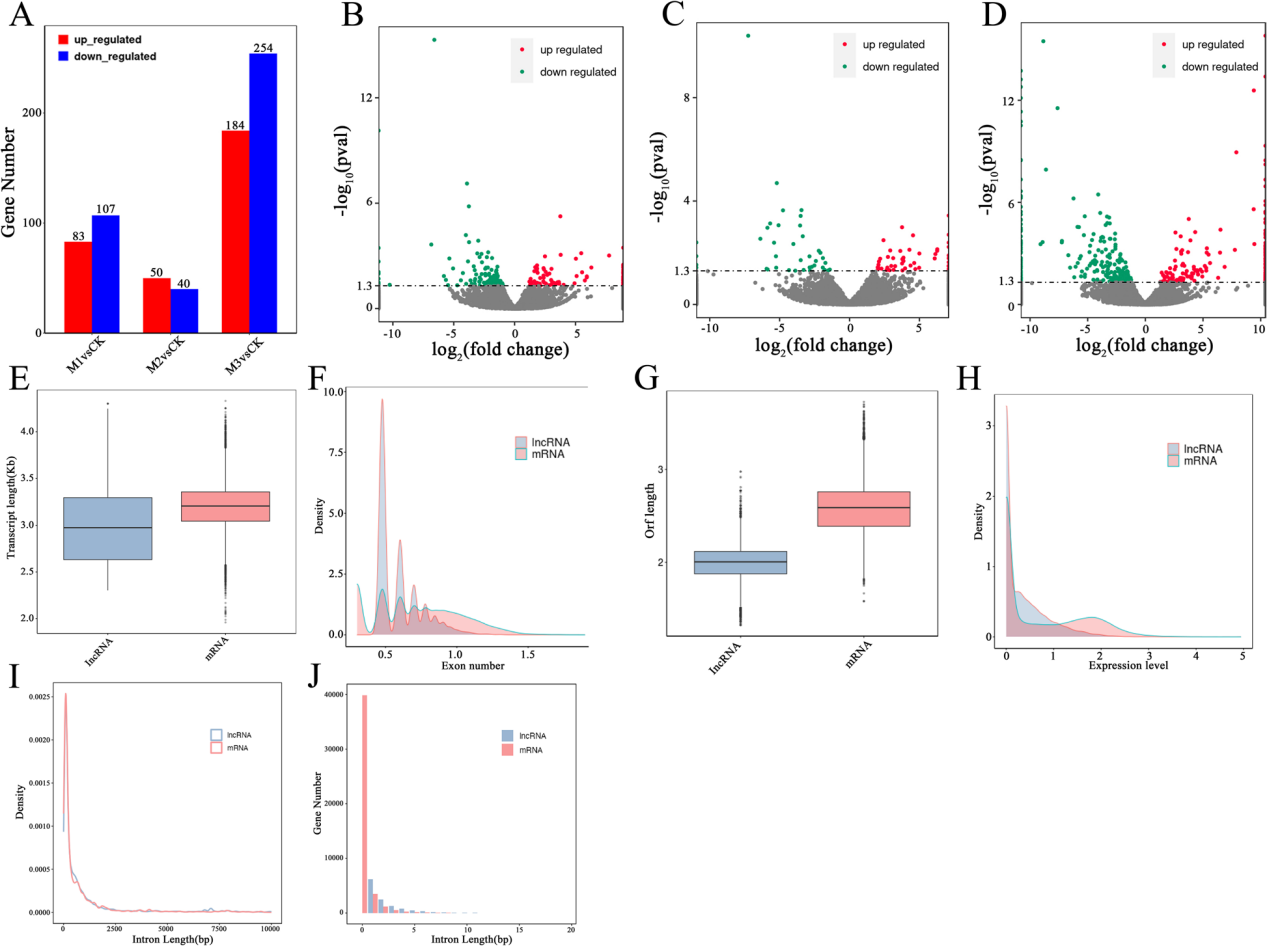
Comparison of lncRNA and mRNAs characteristics in hulless barley. **A** Differentially expressed lncRNA gene number, (**B-D**) Volcano plots of differentially expressed lncRNA genes after mowing at 2 h, 24 h, and 72 h, (**E**) transcript length, (**F**) exon number, (**G**) ORF length, (**H**) expression level, **I** intron length, (**J**) intron number, CK, control check; M1, mowing after 2 h; M2, mowing after 24 h; M3, mowing after 72 h

We compared the length distribution, exon count, open reading frame (ORF) length, and expression levels of the identified novel lncRNAs to protein-coding transcripts to investigate the essential genomic characteristics of these transcripts. The lengths of the identified lncRNAs varied from 200 to 20,070 nucleotides (nt), with an average of 1446 nt. Conversely, protein-coding transcripts ranged from 90 to 21,469 nt in length, averaging1872 nt (Fig. 2E). These finding indicate that lncRNAs are generally shorter than protein-coding transcripts.

Gene structure analysis revealed that lncRNAs, on average, have three exons. However, most lncRNAs (51.18%) possess two exons, with 20 being the maximum (Fig. 2F). In contrast, protein-coding transcripts have an average of five exons, with most mRNAs (16.99%) having only one exon and a maximum going up to 78 exons. However, the average number of exons per lncRNA transcript is 2.3, less than that of protein-coding transcripts (3.4). These findings suggest that, compared to protein-coding transcripts, lncRNAs are often shorter and contain fewer exons, with 2-exons lncRNAs accounting for more than half of the total lncRNA population. The average open reading frame (ORF) length in lncRNAs is 107 nt, ranging from 20 to 946 nt, whereas the average ORF length in mRNAs is 452 nt, ranging from 37 to 5073 nt (Fig. 2G). The number and length of introns are different between lncRNAs and mRNA (Fig. 2I, G).

In terms of expression levels, lncRNAs exhibit distinct patterns compared to mRNAs. FPKM values show 288 mRNAs with expression levels exceeding 1000, while only nine lncRNAs demonstrate such high expression levels. However, it is worth noting that the highest expression value among lncRNAs is 87,359.17, which is more than three times higher than the highest value in mRNAs (27,810.56). These results demonstrate the substantial variations in lncRNA and mRNA expression levels (Fig. 2H). After filtering, 5,749 genes exhibited differential expression between lncRNAs and mRNAs (Fig. 2A).

### Differentially expressed genes (DEGs) after mowing

**Fig. 3:**
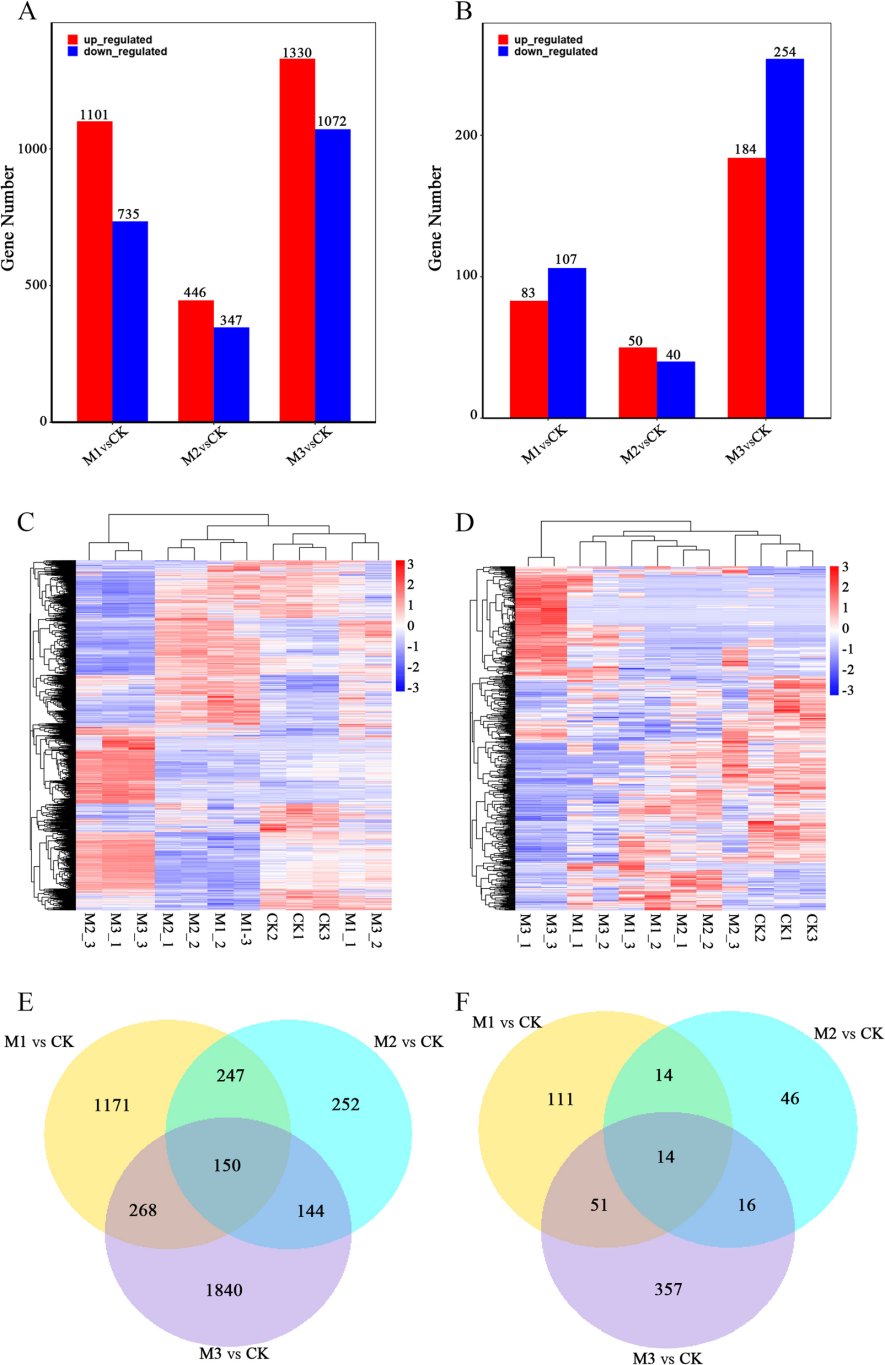
Differentially expressed mRNA and lncRNAs in hulless barley following mowing. **A** DEGs of mRNA. **B** DEGs of lncRNAs. **C** Cluster heat map of differentially expressed mRNAs. **D** Cluster heat map of differentially expressed lncRNAs. **E** Venn diagram showing differentially expressed mRNAs. **F** Venn diagram showing differentially expressed lncRNAs

Following data filtering, 5,031 mRNA genes exhibited differential expression after mowing. The quantity of differentially expressed genes initially decreased but then increased with time post-mowing. The number of differentially expressed genes varied from 793 at 24 h after mowing to 2,402 at 72 h (Fig. 3A). Clustering analysis was used to investigate the expression patterns of these genes at various time points after mowing. Interestingly, these mRNAs showed significant alterations in their expression profiles post-mowing (Fig. 3C). In addition, we evaluated the collective response of mRNAs to mowing treatment, identifying 150 genes (Supplemental Table S1) involved in the mowing reaction at various time points (Fig. 3E).

In contrast to mRNA, 643 differentially expressed lncRNA genes were identified following the mowing treatment (Fig. 3B). Clustering analysis revealed distinct lncRNA expression patterns at various time points post-mowing (Fig. 3D). A joint response analysis revealed 14 genes (Supplemental Table S2) that consistently responded to the mowing treatment (Fig. 3F). These results indicate that relative to mRNA, lncRNAs function as regulatory factors in a smaller subset of genes in response to mowing. However, the 14 lncRNAs identified as having a prolonged response to mowing may play a crucial role in the plant’s recovery after mowing.

### KEGG analysis of target genes regulated by differentially expressed lncRNA

**Fig. 4:**
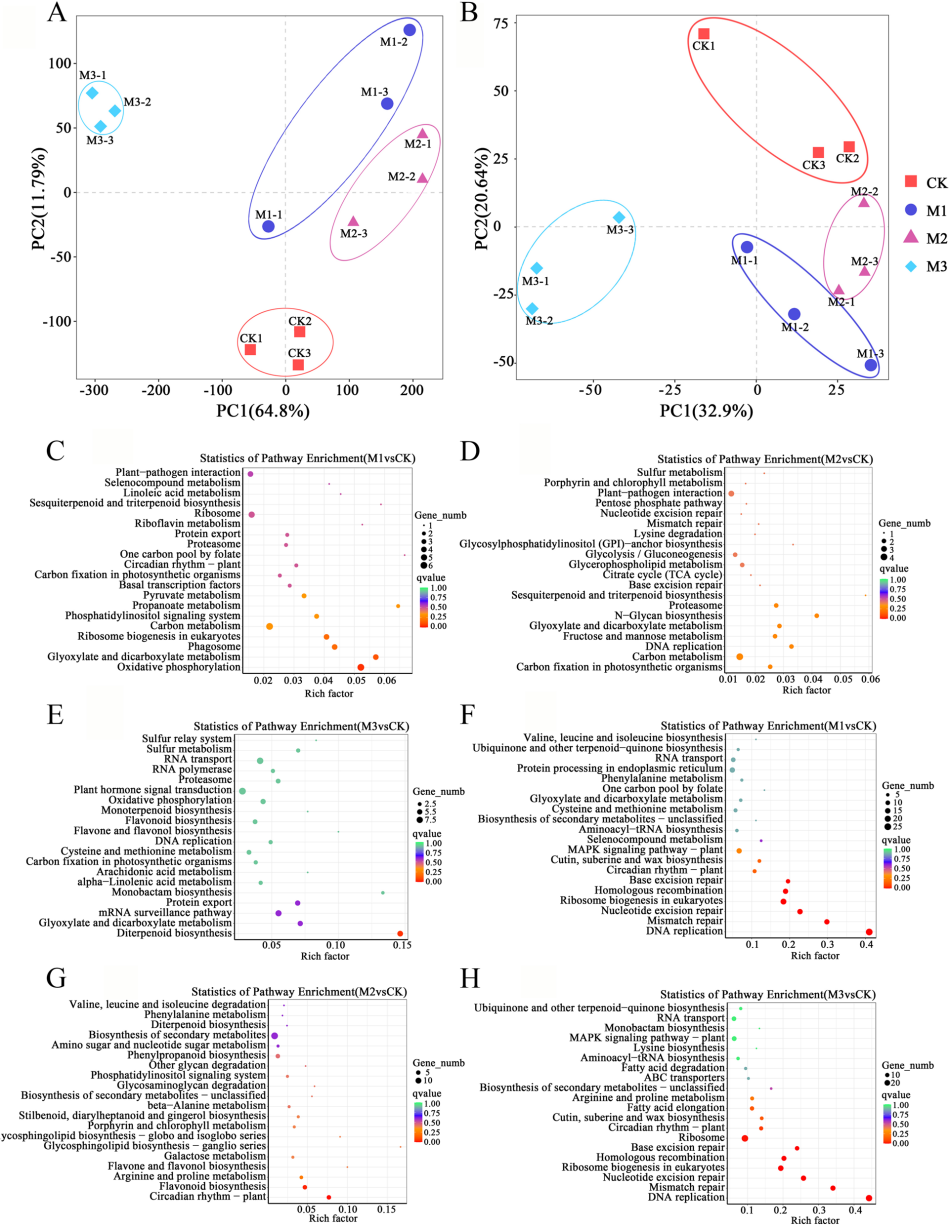
KEGG enrichment analysis of differentially expressed mRNA and lncRNA. **A** PCA analysis of mRNA. **B** PCA analysis of lncRNA. **C-E** KEGG analysis of differential cis-target genes of mRNA in the M1 vs CK, M2 vs CK and M3 vs CK groups, respectively. **F–H** KEGG analysis of differentially expressed trans-target genes of lncRNA in the M1 vs CK, M2 vs CK and M3 vs CK groups, respectively

As crucial regulatory factors, lncRNAs play vital roles in orchestrating essential biological processes such as RNA metabolism and protein activity [20]. We performed PCA modeling analysis on mRNA and lncRNA data at various time points, revealing significant differences between the two groups (Fig. 4A and B). The target mRNA genes that lncRNA regulated after mowing were then subjected to cluster analysis and the analysis of cis- and trans-target mRNA pathways suggested possible pathways that lncRNA might regulate. The cis-target genes were predominantly enriched at two hours post-mowing in pathways associated with oxidative phosphorylation, carbon metabolism, and phagosome and ribosome biogenesis in eukaryotes (Fig. 4C). This suggests that lncRNAs may stimulate the expression of genes related to energy-synthesizing pathways immediately after mowing. At 24 h post-mowing, the enriched pathways of cis-target genes comprised plant-pathogen interaction, carbon metabolism, and glycolysis/gluconeogenesis (Fig. 4D). These findings indicate that 24 h after mowing, plants activate defense mechanisms and increase energy metabolism through pathways, including glycolysis. Diterpenoid biosynthesis was the enriched pathway among cis-target genes at 72 h post-cleavage (Fig. 4E), indicating that the formation of secondary metabolites starts gradually after a certain amount of time post-mowing.

The enrichment analysis of the antisense target genes revealed the predominant enrichment of distinct pathways at various time points following mowing. DNA replication, ribosome biogenesis in eukaryotes, and nucleotide excision repair were the most enriched pathways two hours after mowing (Fig. 4F), indicating the activation of gene expression, repair mechanisms, and ribosome.

At 24 h post-mowing, the primary enrichment was observed in flavonoid biosynthesis and circadian rhythm pathways (Fig. 4G). Similarly, 72 h post-mowing, the enriched pathways resembled those observed at 2 h, with a focus on gene replication, repair, and ribosome synthesis (Fig. 4H). These results provide insights into the temporal dynamics of pathway enrichment in the antisense target genes following mowing, emphasizing the activation of specific biological processes associated with gene expression, repair, and ribosome synthesis at various time points.

### Expression analysis of the mRNA target genes by qRT-PCR

**Fig. 5:**
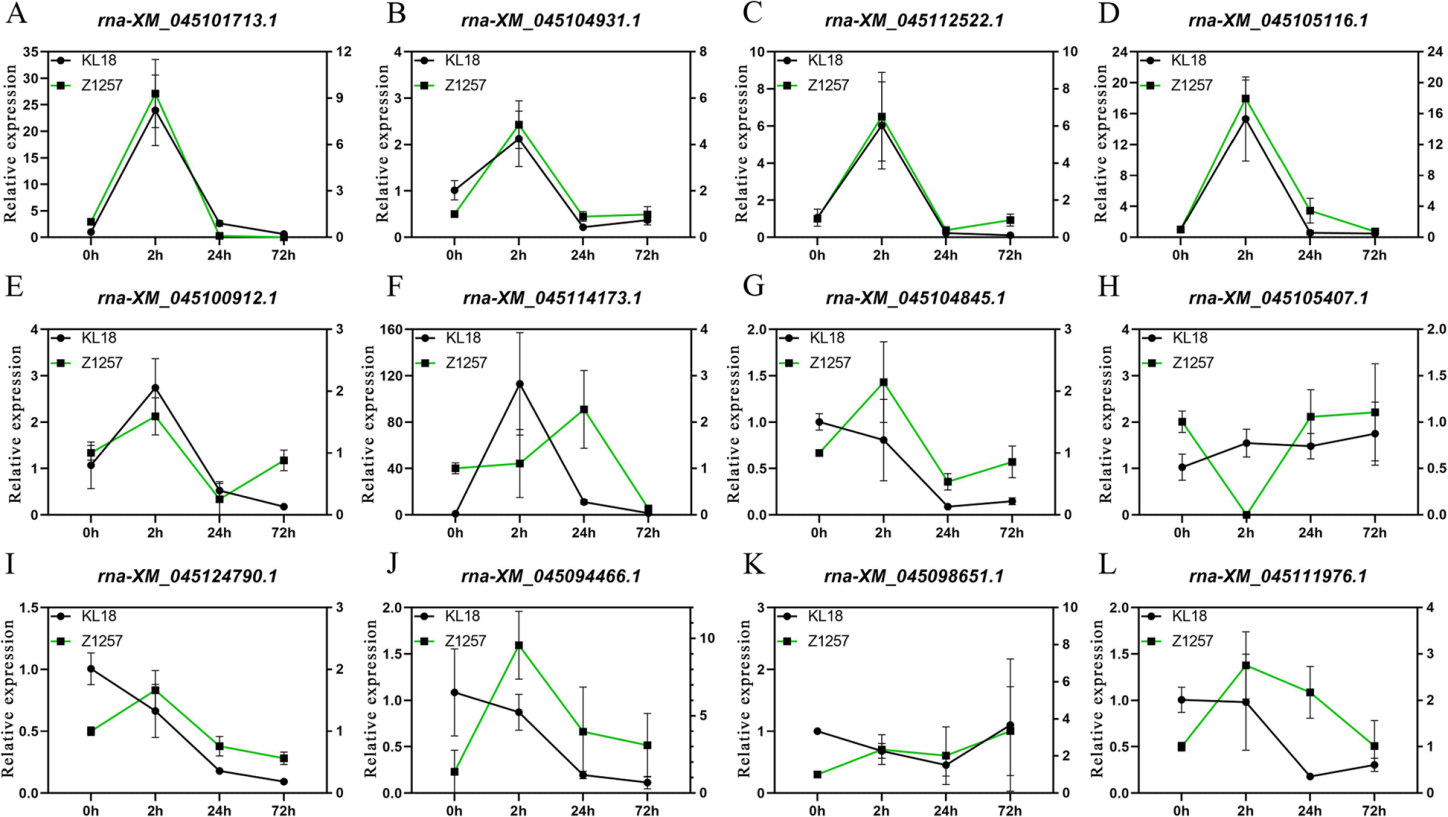
Analysis of mRNA target genes by qRT-PCR. The expression pattern of mRNA target genes in response to mowing is displayed here. The shown data are the mean ± SD of three replicates. 18S rRNA was used as the reference gene

mRNAs are pivotal in regulating target genes that affect plant growth, development, and stress responses. This study determined the target genes of differentially expressed mRNAs implicated in various pathways, including hormone signaling, stress response, and energy metabolism. To investigate the impact of mowing on gene expression, we compared the expression levels of these target genes in two hulless barley varieties: KL18, which recovers quickly, and Z1257, which recovers slowly. Our findings revealed that target genes rna-XM_045101713.1 (L-ascorbate oxidase), rna-XM_045104931.1 (ABC transporter G family member 14-like), rna-XM_045112522.1 (β-fructofuranosidase), and rna-XM_0451051161.1 (TRIUR3_33801 transcription factor) exhibited similar expression patterns after mowing, indicating a coordinated response to the mowing treatment across different hulless barley samples. Conversely, target genes such as rna-XM_045104845.1 (disease resistance protein RGA3 isoform X2), rna-XM_045105407.1 (under abiotic stress and in seed development), rna-XM_045124790.1 (two-component response regulator ARR-B family, response to stimulus), rna-XM_045094466.1 (two-component response regulator ARR-B family, response to stimulus), rna-XM_045100912.1(alpha-glucan water dikinase, chloroplastic-like), rna-XM_045114173.1(NAC domain-containing protein 104), rna-XM_045098651 (*Panicum hallii* probable polygalacturonase), and rna-XM_045111976.1 (REVEILLE1 like gene), exhibited contrasting responses to mowing in different hulless barley varieties. These results demonstrate that mRNA target genes exhibit different expression patterns following mowing treatment in hulless barley varieties with variable recovery abilities (Fig. 5).

### Identification of differential metabolites

**Fig. 6:**
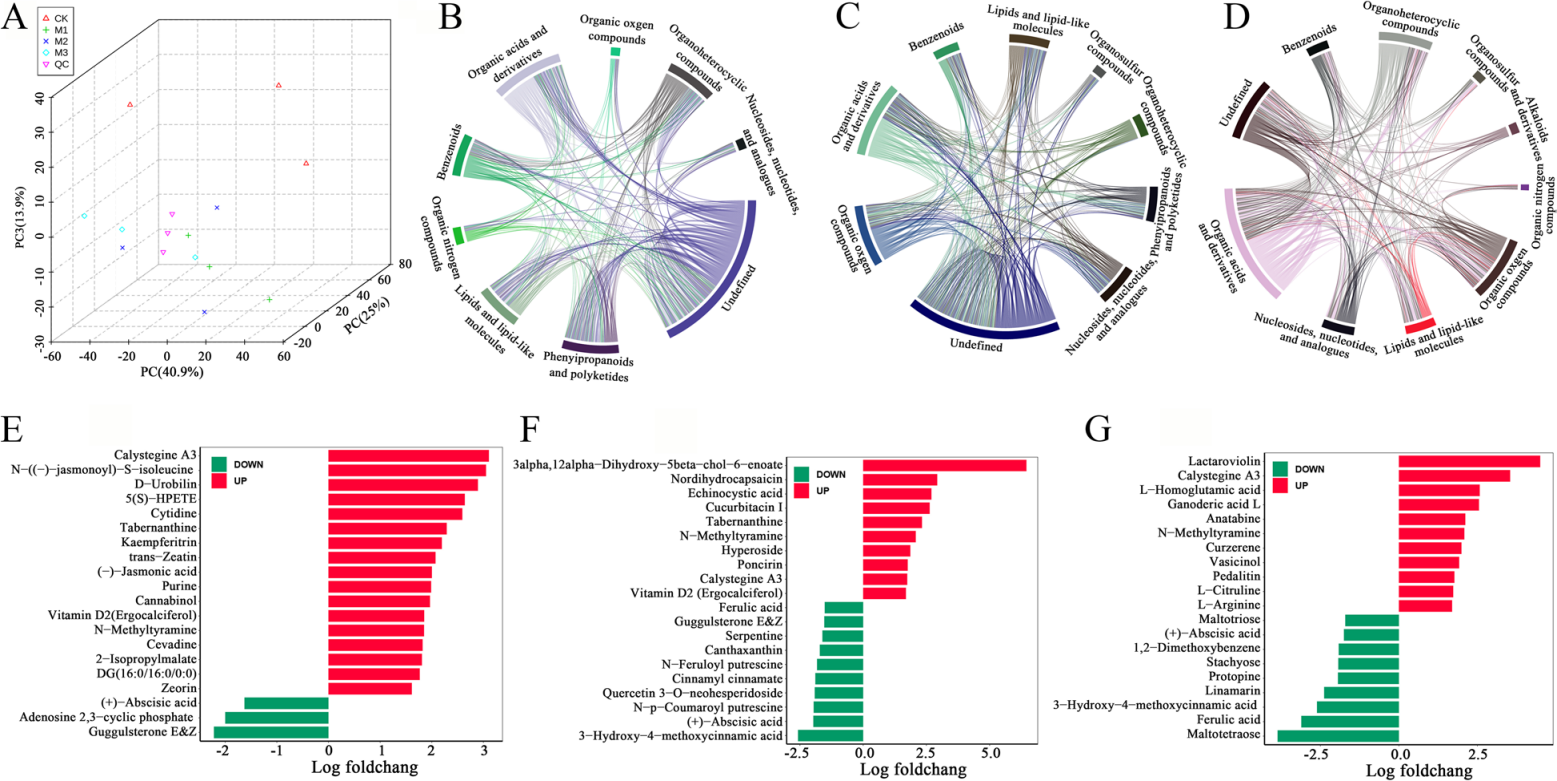
Differential metabolites analysis. **A** PCA analysis. **B-D** Network visualization of differential metabolite molecular pairs in M1 vs CK, M2 vs CK, and M3 vs CK groups, with correlation coefficient |r|> 0.8 and *p* < 0.05. **E–G** The top 20 most significant differential metabolites in M1 vs CK, M2 vs CK, and M3 vs CK groups, respectively

We conducted a non-targeted metabolomics study to investigate the effects of mowing on nutrient accumulation and metabolism in hulless barley. The PCA modeling method revealed significant differences between groups (Fig. 6A). A broad-targeted metabolome yielded 817 compounds. Visualizing correlations between different classes of metabolites at various time points provided a more intuitive understanding (Fig. 6B-D). Differential metabolites were analyzed by sampling at various time points after mowing, taking 0 h as the control group (CK). The analysis yielded 83, 60, and 95 differential metabolites at 2 h, 24 h, and 72 h, respectively. We primarily focused on the top 20 most significant differential metabolites. At two hours, calystegine A3, JA-Ile, JA, and calystegine A3 levels increased, but ABA levels decreased (Fig. 6E). At 24 h, 3alpha, 12alpha − dihydroxy − 5beta − chol − 6 − enoate, nordihydrocapsaicin, echinocystic acid, and other compounds increased, while ABA and methoxycinnamic acid levels showed a decline (Fig. 6F). Lactaroviolin and calystegine A3 accumulated after 72 h, but ABA and certain maltotetraose-like compounds decreased (Fig. 6G).

### KEGG analysis of differential metabolites

**Fig. 7:**
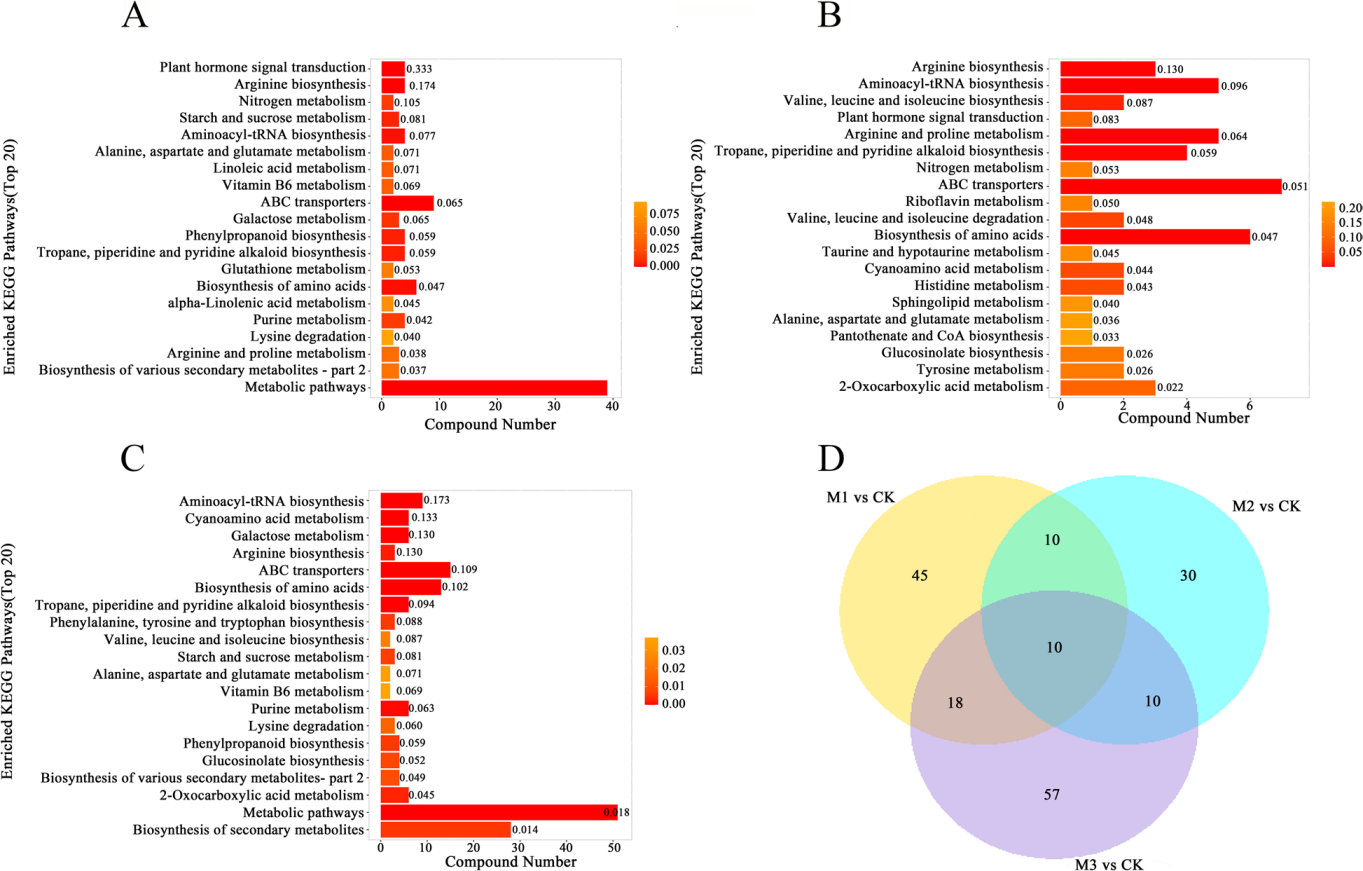
KEGG analysis of differential metabolites. **A-C** KEGG analysis of differential metabolites in the M1 vs CK, M2 vs CK, and M3 vs CK groups, respectively. **D** Venn diagram of differential metabolites after mowing

We conducted a KEGG analysis of the differential metabolites following mowing. Fig. 7A shows the results of the 2-h post-mowing group, versus the control group. The differential metabolites between the two groups are predominantly enriched in metabolic pathways, including amino acid biosynthesis, ABC transporters, and hormonal pathways. Fig. 7B shows the results of the 24-h post-mowing group against the control group, with differential metabolites mainly enriched in amino acid synthesis pathways. Fig. 7C compares the results of the 72-h post-mowing group to the control group, revealing that the differential metabolites were primarily enriched in metabolic pathways, including biosynthesis of secondary metabolites and starch and sugar metabolism. The Venn diagram of differential metabolites shows that ten metabolites varied significantly across all groups (Fig. 7D). Four metabolites, including calystegine A3, accumulated, while six compounds, including ABA, declined after mowing (Supplemental Table S3).

### The response of phytohormones to mowing

**Fig. 8:**
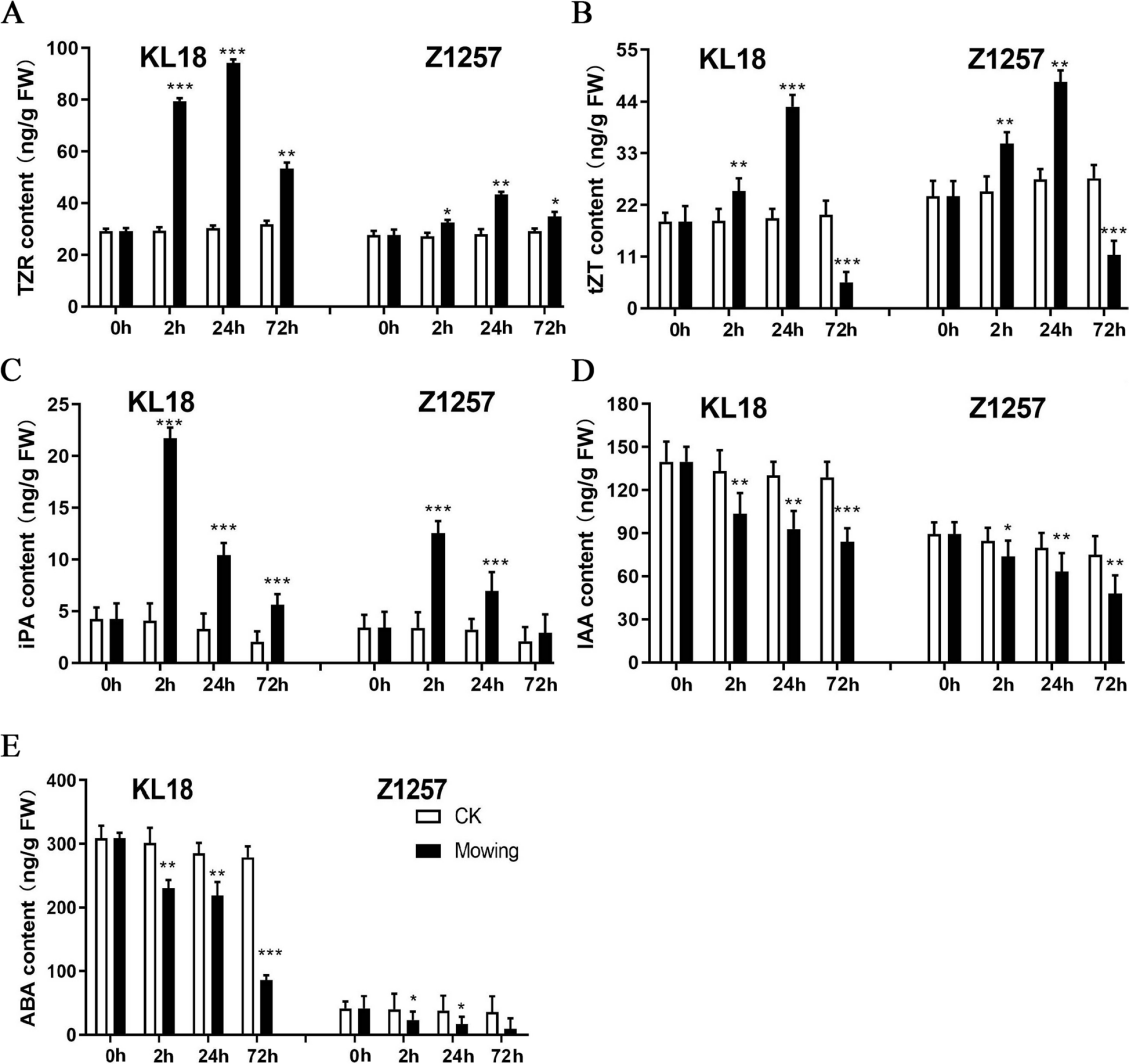
Hormones content in hulless barley after mowing at 0 h, 2 h, 24 h, and 72 h. **A** TZR content, (**B**) tZT content, (**C**) iPA content, (**D**) IAA content, (**E**) ABA content. Values are means ± SD (*n* = 3) (*P ≤ 0.05, **P ≤ 0.01, **P ≤ 0.001, Student’s *t*-test)

Plant hormones play a crucial role in regulating plant growth and development. The metabolomic analysis revealed significant variations in hormonal pathways between the control (CK) and mowing groups. Thus, we investigated the response of trans-Zeatin-riboside (TZR), Trans-Zeatin (tZT), isopentenyl adenosine (iPA), Indole-3-acetic acid (IAA), and abscisic acid (ABA) in the KL18 and Z1257 varieties following mowing. Our findings demonstrated that the KL18 and Z1257 varieties produced considerably more TZR after mowing. Notably, the accumulation of TZR in the KL18 variety reached 94.22 ng/g FW, which was 3.11 times higher than that in the CK group (Fig. 8A). Similarly, mowing increased the accumulation of tZT in the Z1257 variety by up to 1.55 times than that of CK (Fig. 8B). In both varieties, tZT initially increased significantly after mowing but decreased after 72 h. iPA also significantly induced in both the KL18 and Z1257 varieties (Fig. 8C). However, the concentration of iPA induced by mowing in KL18 was particularly notable, as it reached 21.72 ng/g FW, 5.33 times higher than CK group. Similarly, the concentration of iPA induced by mowing in Z1257 reached a maximum of 12.55 ng/g FW, representing a 3.71-fold increase over the CK (Fig. 8D). In contrast, both IAA and ABA levels decreased significantly after mowing. The constitutive concentrations of these two hormones were lower in the Z1257 variety than in KL18, with the ABA content being exceptionally low at 38.95 ng/g FW in Z1257 and 293.76 ng/g FW in KL18 (Fig. 8E). These results highlight the crucial role that plant hormones play in the growth and development of hulless barley following mowing.

## Discussion

Mowing hulless barley provides a valuable food source for both people in pastoral areas and livestock [21]. Previous research on hulless barley has focused on stress resistance, nutritional quality, and improving wheat quality [22–24]. However, the mechanisms governing nutrient accumulation, growth, and development after mowing in hulless barley remain unknown. LncRNAs are sometimes referred to as “dark matter” or “transcriptional noise,” although they have recently been discovered to serve crucial regulatory roles in plant growth, development, and stress resistance [25, 26]. Mowing is a form of damaging stress that shares characteristics with pathogen invasion. LncRNAs regulate plant resistance mediated by JA, enhancing resistance to herbivores [27]. For instance, MISSEN, a lncRNA expressed in early rice endosperm, impacts seed development by regulating microtubule protein-mediated cytoskeleton assembly [28]. Biological stress, such as aphid damage to plants, is comparable to mowing damage. Aphids puncture leaves to extract sap, triggering the expression of many lncRNAs [29]. In addition, lncRNAs in animal skin cells promote glial cell formation and migration, which regulates wound healing [30, 31]. sRNAs and lncRNAs belong to non-coding RNAs that can regulate plant growth and development. sRNAs can produce quickly and regulate genes expression across species [40], while there is no reported that lncRNA regulate genes expression across species. In addition, lncRNAs may be the potential targets of sRNAs, sRNAs can regulate genes expression by regulating the expression of lncRNA [41]. Therefore, there must be interaction between sRNA and lncRNA in when hulless barley responds to mowing. However, the mechanism of how small RNAs and lncRNAs interact with each other to affect the recovery of hulless barley regeneration is unclear, further research still needed in this direction.

In this study, we used transcriptome profiling to identify differentially expressed mRNA and lncRNA genes in hulless barley at various time points post-mowing. Our analysis revealed 150 differentially expressed mRNA genes and 14 differentially expressed lncRNA genes (Fig. 3C and F).

KEGG analysis of the various time points indicated that the pathways shared between the 2-h and 24-h time points had the most overlap. These pathways included cysteine and methionine metabolism, porphyrin and chlorophyll metabolism, plant-pathogen interaction, carotenoid biosynthesis, carbon fixation in photosynthetic organisms, glyoxylate and dicarboxylate metabolism, circadian rhythm, photosynthesis, and photosynthesis-antenna proteins. This indicates that the defense system, chlorophyll metabolism, photosynthesis, and the methylerythritol phosphate pathway were primarily affected before the 24-h mark (Supplemental Fig. 1). Mowing consistently impacted the photosynthesis-antenna proteins pathway across all three time points, indicating its long-term consequences on photosynthesis (Supplemental Fig. 1). Further KEGG analysis of cis-regulated lncRNA target genes revealed enrichment of carbon fixation in photosynthetic organisms and carbon metabolism in both the 2-h and 24-h datasets, suggesting that carbon fixation in photosynthesis responds quickly to the energy demand induced by mowing. Proteasome, glyoxylate, and dicarboxylate metabolism pathways were consistently enriched at different time points (Fig. 4C), indicating that material degradation metabolism provides energy for post-mowing plant growth and development.

In addition, KEGG analysis of trans-regulated lncRNA target genes revealed enrichment of the phenylalanine metabolism pathway at 2- and 24-h time points. In contrast, at all three time points, there was a consistent enrichment of circadian rhythm and secondary metabolite biosynthesis pathways (Fig. 4F). Overall, the analysis of lncRNA target genes revealed the enrichment of eight pathways: proteasome, circadian rhythm, biosynthesis of secondary metabolites, glyoxylate, and dicarboxylate metabolism, carbon fixation in photosynthetic organisms, base excision repair, mismatch repair, and DNA replication. These findings indicate a coordinated response to mowing stress involving carbon synthesis, protease pathways, base replication and repair, and rhythmic gene regulation.

Plant metabolite accumulation affects the nutritional value of the plants as well as their flavor as feed [32, 33]. Metabolomic analysis of hulless barley at various time points after mowing revealed four differentially accumulated metabolites with increased and six with decreased levels (Supplemental Table S3). The accumulated metabolites include calystegine A3, L-arginine, L-citrulline, and N-methyltyramine. Prior studies have indicated that calystegine can function as a nitrogen and carbon source for beneficial microorganisms within the plant rhizosphere, consequently promoting plant growth [34]. In addition, elevated levels of L-arginine and L-citrulline augment the availability of amino acids in forage grass. Higher levels of N-methyltyramine can stimulate animal appetite, improve nutrient absorption, inhibit degradation, and increase energy intake [35]. N-methyltyramine also improves gastrointestinal conditions in animals [36]. The six decreased metabolites include diosmetin, ferulic acid, lactulose, abscisic acid, turanose, and 3-hydroxy-4-methoxycinnamic acid. Reducing ferulic acid, a phenolic compound, may enhance the palatability of hulless barley as feed. Under standard settings, a drop in abscisic acid (ABA) content is known to boost plant growth [37], implying that reducing ABA content may benefit plant growth. Diosmetin, a flavonoid, inhibits cell proliferation by suppressing the cell cycle and interfering with lipid metabolism [38]. Turanose is a non-metabolizable plant sugar that can act as a sugar signal and limit lipid accumulation [39].

To investigate the relative variations in metabolite abundance across various samples, we standardized and centered the relative abundance of the differentially identified metabolites before performing K-means clustering analysis (Supplemental Fig. 2). Some subclusters showed exciting trends. For instance, subcluster_4 had metabolites with increasing abundance following mowing and included DL-tyrosine, L-histidine, L-tyrosine, L-valine, and pyridoxine (Supplemental Fig. 2d). Subcluster_12, including 13-HPODE (a lipid peroxide), salicylic acid, syringic acid, and methyl linoleate, showed an initial increase followed by a subsequent drop (Supplemental Fig. 2 l). These observed trends provide insights into the dynamic changes in metabolites after mowing, with the continuous accumulation of amino acids constituting the most direct response. Besides metabolite content will be regulated by the feedback of upstream and downstream metabolites [42, 43]. The mechanism of feedback regulation among metabolites during regeneration and recovery of hulless barley plants remains to be further investigated.

Plant hormones play a critical role in plant development and stress responses. We measured CTK, IAA, and ABA in two hulless barley varieties with contrasting growth rates following mowing. The Z1257 variety exhibited slower growth and a significantly lower ABA content of 38.95 ng/g FW compared to KL18, which had a content of 293.76 ng/g FW before mowing. Similarly, the IAA content in Z1257 is 82.22 ng/g FW, lower than the 133.1 ng/g FW observed in KL18. Before mowing, the Z1257 variety had a higher tZT level of 25.92 ng/g FW than KL18, which had a content of 18.99 ng/g FW (Supplemental Fig. 3). The ABA content in KL18 was significantly lower at 72 h after mowing, whereas there was no significant difference in Z1257 compared to the control (Fig. 8E).

Given that ABA inhibits plant growth, the rapid recovery of ABA concentration after mowing may contribute to the slow growth observed in the Z1257 variety. In addition, mowing increased the levels of cytokinins TZR, tZT, and iPA in KL18 compared to Z1257. iPA showed a significant spike in the KL18 variety 72 h after mowing compared to the control (Fig. 8A). These findings suggest that CTKs and ABA play essential functions in the regeneration of hulless barley following mowing.

It is also common practice to use LncRNA databases for analysis; for example, in rice [45–49], maize [50], wheat [53], peanut [44], sunflower [52], and cereal [51], lncRNAs have been reported in relation to the regulation of their key traits. These findings offer novel insights into regulating lncRNAs and the metabolite changes during the growth and development of hulless barley following mowing. Our comprehensive genome-wide identification and functional analysis of lncRNAs, combined with the characterization of metabolite changes post-mowing, will lay a robust theoretical foundation for future research into the molecular mechanisms underlying mowing in hulless barley and other plants.

## Conclusion

This study established a comprehensive dataset of lncRNAs, metabolites, and hormones in hulless barley after mowing, providing valuable insights into these components’ roles in regulating plant regeneration. Specifically, the results highlight cytokinin’s role in facilitating the regeneration process of hulless barley after mowing. This comprehensive dataset is a valuable tool for further understanding the complex mechanisms underlying plant regeneration, which may affect crop improvement strategies.

### Supplementary Information

Supplementary Material 1.

Supplementary Material 2.

## Data Availability

The datasets generated and/or analysed during the current study are available in the NCBI (https://www.ncbi.nlm.nih.gov/) Sequence Read Archive repository with accession number: PRJNA1085841.

## References

[1] Pankin A, von Korff M (2017). Co-evolution of methods and thoughts in cereal domestication studies: a tale of barley (Hordeum vulgare). Curr Opin Plant Biol.

[2] Zeng X, Guo Y, Xu Q, Mascher M, Guo G, Li S (2018). Origin and evolution of qingke barley in Tibet. Nat Commun.

[3] Guo T, Horvath C, Chen L, Chen J, Zheng B (2020). Understanding the nutrient composition and nutritional functions of highland barley (Qingke): a review. Trends Food Sci Technol.

[4] Liu H, Li Y, You M, Liu X (2021). Comparison of physicochemical properties of β-glucans extracted from hull-less barley bran by different methods. Int J Biol Macromol.

[5] WU K-L, Yao X-H, Yao Y-H, Bai Y-X, Chi D-Z. Reflections and practice on breeding barley varieties under the background of diversified uses. Tibet J Agri Sci. 2018;40(S1):1–2.

[6] Cui Z-M, Guo G, Yuan X-J, LI J-F, Yang X-D, Ding L (2015). Characterization and identification of high quality lactic acid bacteria from hulless barley straw silage. Acta Agrestia Sinica.

[7] Anfang C, Schuster MJ, Wragg PD, Reich PB (2020). Increased light availability due to forestry mowing of invasive European buckthorn promotes its regeneration. Restor Ecol.

[8] Wang M, Xu Z, Song J, Liu X, Jiao X (2018). Effects of different mowing treatments and stubble heights on the compensatory growth and quality of lettuce (Lactuca sativa L.). J Horticultural Sci Biotechnol..

[9] Kulik M, Sender J, Bochniak A, Jaźwa M, Ciesielski D (2023). The influence of mowing frequency on the growth and development of phragmites australis. J Nat Conserv.

[10] Zhou J, Wilson GW, Cobb AB, Yang G, Zhang Y (2019). Phosphorus and mowing improve native alfalfa establishment, facilitating restoration of grassland productivity and diversity. Land Degrad Dev.

[11] Smith AL, Barrett RL, Milner RN (2018). Annual mowing maintains plant diversity in threatened temperate grasslands. Appl Veg Sci.

[12] Yang Z, Minggagud H, Baoyin T, Li FY (2020). Plant production decreases whereas nutrients concentration increases in response to the decrease of mowing stubble height. J Environ Manage.

[13] Zhang Y, Yin Y, Amombo E, Li X, Fu J (2020). Different mowing frequencies affect nutritive value and recovery potential of forage bermudagrass. Crop Pasture Sci.

[14] Li S, Wang S, Ye W, Yao Y, Sun F, Zhang C (2023). Effect of mowing on wheat growth at seeding stage. Int J Mol Sci.

[15] Zhou R, Sanz-Jimenez P, Zhu X-T, Feng J-W, Shao L, Song J-M (2021). Analysis of rice transcriptome reveals the lncRNA/circRNA regulation in tissue development. Rice.

[16] Wu L, Liu S, Qi H, Cai H, Xu M (2020). Research progress on plant long non-coding RNA. Plants.

[17] Cui G, Zhao M, Tan H, Wang Z, Meng M, Sun F (2021). RNA sequencing reveals dynamic carbohydrate metabolism and phytohormone signaling accompanying post-mowing regeneration of forage winter wheat (Triticum aestivum L.). Front Plant Sc..

[18] Nokhsorov VV, Dudareva LV, Semenova NV, Petrov KA (2023). Study of the Effect of Mowing and Drying on the lipid composition of grass leaves in permafrost ecosystems. Agronomy.

[19] Livak KJ, Schmittgen TD (2001). Analysis of relative gene expression data using real-time quantitative PCR and the 2− ΔΔCT method. Methods..

[20] Yu Y, Zhang Y, Chen X, Chen Y (2019). Plant noncoding RNAs: hidden players in development and stress responses. Annu Rev Cell Dev Biol.

[21] Alazmani A (2015). Effect of sowing dates and population on yield and yield components and forage in dual purpose cultivation of hulless barley (Hordeum vulgare L.). J Adv Bot Zool..

[22] Narwal S, Kumar D, Sheoran S, Verma RPS, Gupta R (2017). Hulless barley as a promising source to improve the nutritional quality of wheat products. J Food Sci Technol.

[23] Yangcheng H, Gong L, Zhang Y, Jane J-I (2016). Pysicochemical properties of Tibetan hull-less barley starch. Carbohydr Polym.

[24] Du J-B, Yuan S, Chen Y-E, Sun X, Zhang Z-W, Xu F (2011). Comparative expression analysis of dehydrins between two barley varieties, wild barley and Tibetan hulless barley associated with different stress resistance. Acta Physiol Plant.

[25] Jha UC, Nayyar H, Jha R, Khurshid M, Zhou M, Mantri N (2020). Long non-coding RNAs: emerging players regulating plant abiotic stress response and adaptation. BMC Plant Biol.

[26] Meng X, Li A, Yu B, Li S (2021). Interplay between miRNAs and lncRNAs: Mode of action and biological roles in plant development and stress adaptation. Comput Struct Biotechnol J.

[27] Li R, Jin J, Xu J, Wang L, Li J, Lou Y (2021). Long non-coding RNAs associate with jasmonate-mediated plant defence against herbivores. Plant, Cell Environ.

[28] Zhou Y-F, Zhang Y-C, Sun Y-M, Yu Y, Lei M-Q, Yang Y-W (2021). The parent-of-origin lncRNA MISSEN regulates rice endosperm development. Nat Commun.

[29] Zhang J, Yang Z, Feng P, Zhong X, Ma Q, Su Q (2019). Identification and the potential roles of long non-coding RNAs in cotton leaves damaged by Aphis gossypii. Plant Growth Regul.

[30] Zhang L, Hu J, Meshkat BI, Liechty KW, Xu J (2021). LncRNA MALAT1 modulates TGF-β1-induced EMT in keratinocyte. Int J Mol Sci.

[31] Li D, Kular L, Vij M, Herter EK, Li X, Wang A (2019). Human skin long noncoding RNA WAKMAR1 regulates wound healing by enhancing keratinocyte migration. Proc Natl Acad Sci.

[32] Kilcawley KN, Faulkner H, Clarke HJ, O’Sullivan MG, Kerry JP (2018). Factors influencing the flavour of bovine milk and cheese from grass based versus non-grass based milk production systems. Foods.

[33] Schreurs N, Lane G, Tavendale M, Barry T, McNabb W (2008). Pastoral flavour in meat products from ruminants fed fresh forages and its amelioration by forage condensed tannins. Anim Feed Sci Technol.

[34] Tepfer D, Goldmann A, Pamboukdjian N, Maille M, Lepingle A, Chevalier D (1988). A plasmid of Rhizobium meliloti 41 encodes catabolism of two compounds from root exudate of Calystegium sepium. J Bacteriol.

[35] Stohs SJ, Hartman MJ (2015). A review of the receptor binding and pharmacological effects of N-methyltyramine. Phytother Res.

[36] Ni J, Guo Y, Chang N, Cheng D, Yan M, Jiang M (2019). Effect of N-methyltyramine on the regulation of adrenergic receptors via enzymatic epinephrine synthesis for the treatment of gastrointestinal disorders. Biomed Pharmacother.

[37] Brookbank BP, Patel J, Gazzarrini S, Nambara E (2021). Role of basal ABA in plant growth and development. Genes.

[38] Pan L, Feng F, Wu J, Li L, Xu H, Yang L (2021). Diosmetin inhibits cell growth and proliferation by regulating the cell cycle and lipid metabolism pathway in hepatocellular carcinoma. Food Funct.

[39] Park M-O, Lee B-H, Lim E, Lim JY, Kim Y, Park C-S (2016). Enzymatic process for high-yield turanose production and its potential property as an adipogenesis regulator. J Agric Food Chem.

[40] De Mets F, Van Melderen L, Gottesman S (2019). Regulation of acetate metabolism and coordination with the TCA cycle via a processed small RNA. Proc Natl Acad Sci.

[41] Yang W, Bai Q, Li Y, Chen J, Liu C (2023). Epigenetic modifications: allusive clues of lncRNA functions in plants. Comput Struct Biotechnol J.

[42] Paul MJ, Pellny TK (2003). Carbon metabolite feedback regulation of leaf photosynthesis and development. J Exp Bot.

[43] Liu T, Kawochar MA, Liu S, Cheng Y, Begum S, Wang E (2023). Suppression of the tonoplast sugar transporter, StTST3. 1, affects transitory starch turnover and plant growth in potato. Plant J..

[44] Ma X, Zhang X, Traore SM, Xin Z, Ning L, Li K (2020). Genome-wide identification and analysis of long noncoding RNAs (lncRNAs) during seed development in peanut (Arachis hypogaea L.). BMC Plant Biol..

[45] Huang X, Zhang H, Wang Q, Guo R, Wei L, Song H (2021). Genome-wide identification and characterization of long non-coding RNAs involved in flag leaf senescence of rice. Plant Mol Biol.

[46] Li M, Cao A, Wang R, Li Z, Li S, Wang J (2020). Genome-wide identification and integrated analysis of lncRNAs in rice backcross introgression lines (BC 2 F 12). BMC Plant Biol.

[47] Leng Y, Sun J, Wang J, Liu H, Zheng H, Zhang M (2020). Genome-wide lncRNAs identification and association analysis for cold-responsive genes at the booting stage in rice (Oryza sativa L.). Plant Genome..

[48] Li L, Guo N, Liu T, Yang S, Hu X, Shi S (2023). Genome-wide identification and characterization of long non-coding RNA in barley roots in response to piriformospora indica colonization. Plant Sci.

[49] He H, Zhou Y-F, Yang Y-W, Zhang Z, Lei M-Q, Feng Y-Z (2021). Genome-wide analysis identified a set of conserved lncRNAs associated with domestication-related traits in rice. Int J Mol Sci.

[50] Cao W, Wang R, Cao J, Gao J, Zhao X, Gan L (2022). Genome-wide identification and characterization of long noncoding RNAs in maize under rice black streaked dwarf virus infection. Plant Pathol.

[51] Zhao Z, Liu D, Cui Y, Li S, Liang D, Sun D (2020). Genome-wide identification and characterization of long non-coding RNAs related to grain yield in foxtail millet [Setaria italica (L.) P. Beauv.]. BMC Genomics.

[52] Yu S, Zhang Z, Li J, Zhu Y, Yin Y, Zhang X (2022). Genome-wide identification and characterization of lncRNAs in sunflower endosperm. BMC Plant Biol.

[53] Zheng W, Hu H, Lu Q, Jin P, Cai L, Hu C (2021). Genome-wide identification and characterization of long noncoding rnas involved in chinese wheat mosaic virus infection of nicotiana benthamiana. Biology.

