# Supplementary material for: Identification and functional analysis of long non-coding RNA (lncRNA) and metabolites response to mowing in hulless barley (Hordeum vulgare L. var. nudum hook. f.)
Source: BMC Plant Biol. 2024 Jul 12;24:666. doi: 10.1186/s12870-024-05334-8 (PMC11241897; doi:10.1186/s12870-024-05334-8)
Supplement: Supplementary file 2 — Supplementary Material 2. [file 12870_2024_5334_MOESM2_ESM.docx]

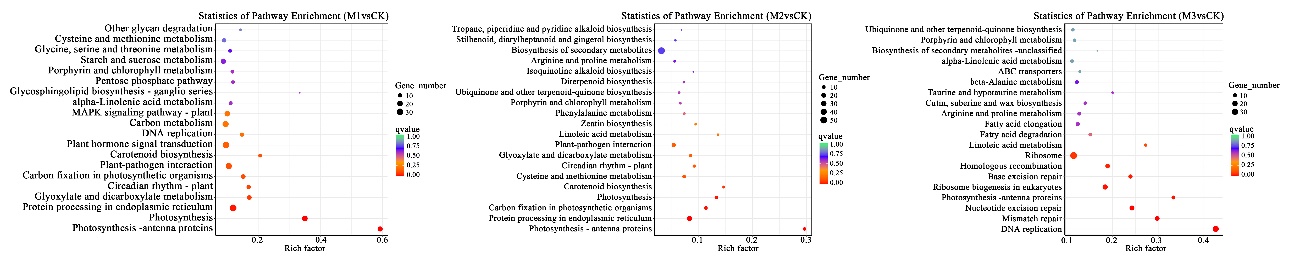


Supplemental Figure 1. (A-C) KEGG pathway enrichment analysis of differentially expressed mRNA in the M1 vs CK, M2 vs CK and M3 vs CK groups, respectively.
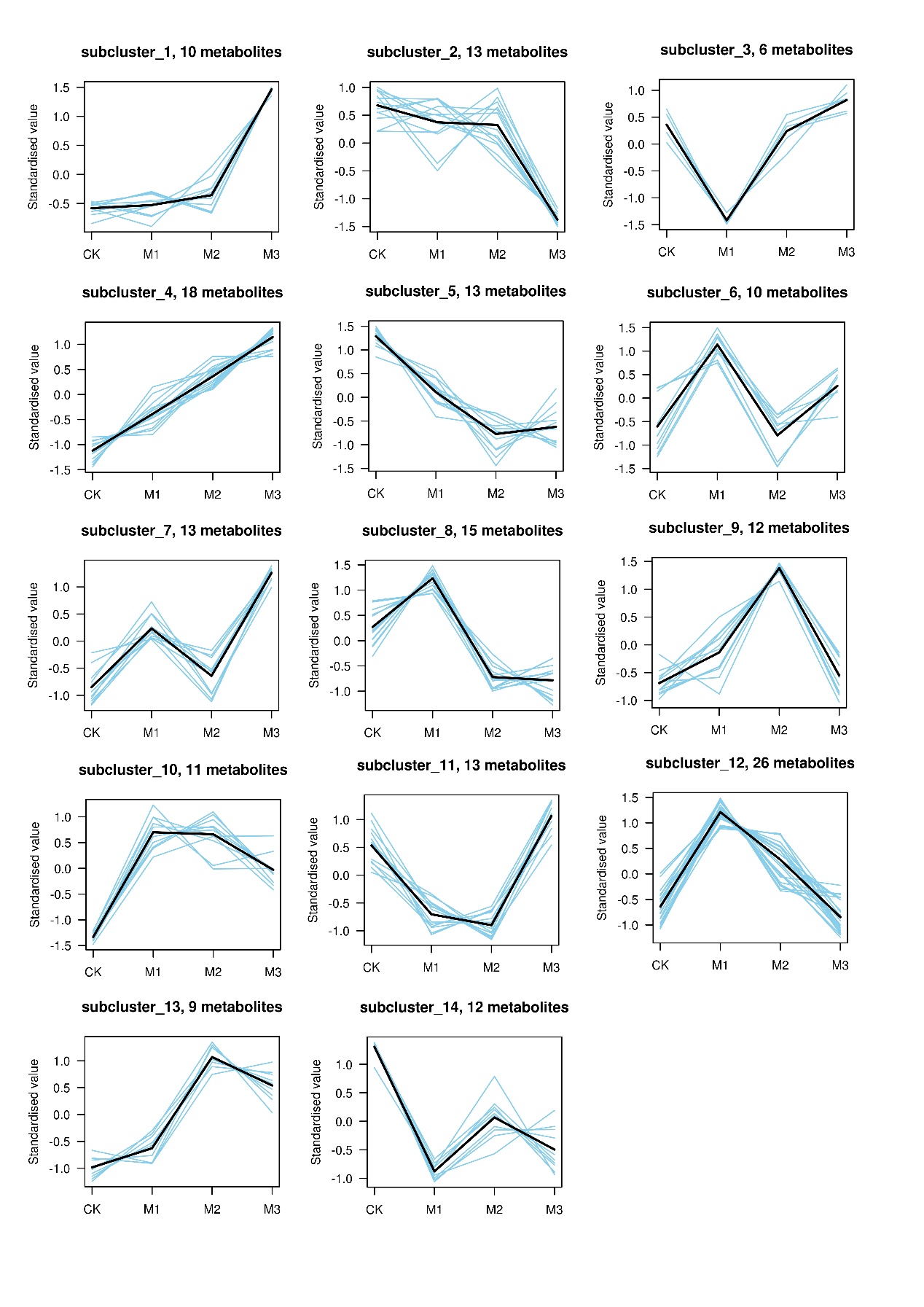


Supplemental Figure 2. (a-n) K-means analysis of differential metabolites trends in the M1 vs CK, M2 vs CK and M3 vs CK groups, respectively.


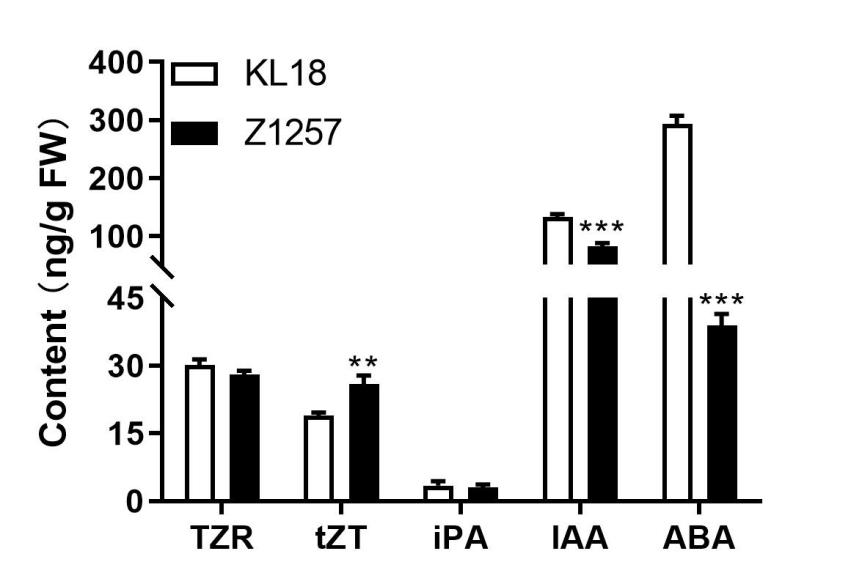


Supplemental Figure 3. Quantification of hormone content (TZR, tZT, iPA, IAA and ABA)

in hulless barley (KL18 and Z1257 varieties) before mowing.
